# Plant Diversity and Seasonal Variation Drive Animal Diversity and Community Structure in Eastern China

**DOI:** 10.3390/ani16020215

**Published:** 2026-01-11

**Authors:** Xiangxiang Chen, Runhan Jiang, Yunhan Chen, Rui Yang, Yan He, Shuai Zou, Jianping Ying, Lixiao Yi, Yuxin Ye, Sili Peng, Zhiwei Ge

**Affiliations:** 1 Co-Innovation Center for Sustainable Forestry in Southern China, College of Ecology and Environment, Nanjing Forestry University, Nanjing 210037, China; xiangxchen@njfu.edu.cn (X.C.); 1156573541@njfu.edu.cn (R.J.); cyh1326594912@outlook.com (Y.C.); yangrui97482@njfu.edu.cn (R.Y.); heyan@njfu.edu.cn (Y.H.); zoushuai1618@126.com (S.Z.); pengsili@njfu.edu.cn (S.P.); 2 Longyou County Forest Resources Monitoring Station, Quzhou 324000, China; cinci1983@163.com; 3 Longyou County Forestry Technology Extension Station, Quzhou 324000, China; ylx8358@163.com; 4 Miaoxia Township People’s Government of Longyou County, Quzhou 324000, China; 17858563031@163.com

**Keywords:** infrared-triggered camera, community structure, multidimensional diversity, seasonal change, altitudinal migration

## Abstract

Montane forests, characterized by complex terrain and diverse climates, serve as critical global biodiversity hotspots, particularly for birds and mammals. However, the patterns and underlying processes of bird and mammal diversity remain insufficiently studied in the montane forests of eastern China. This study employed infrared-triggered camera trapping to conduct a four-year field monitoring of birds and mammals, analyzing the effects of plant diversity and seasonal variations on the diversity of habitat-associated animals. Our results revealed that species-level habitat visit frequency in ground-dwelling birds exhibited a significant phylogenetic signal, particularly in spring and summer. Plant diversity metrics demonstrated significant positive correlations with corresponding bird metrics of species richness (SR), phylogenetic diversity (PD), and the standardized effect size of PD (Phylo SES PD). In contrast, for mammals, plant diversity metrics were significantly positively correlated with corresponding SR, mean pairwise phylogenetic distance (Phylo MPD), and mean nearest phylogenetic taxon distance (Phylo MNTD), as well as community structure metrics, including the net relatedness index (Phylo NRI) and nearest taxon index (Phylo NTI). Furthermore, the plant Shannon–Wiener index showed significant positive correlations with both bird and mammal metrics of SR, PD, and Phylo SES PD but significant negative correlations with Phylo MNTD. Seasonal variations triggered the mean altitudinal migration in ground-dwelling birds and mammals. There were significant differences in the diversity and community structure metrics of birds (Shannon–Wiener, Funct FNND, and PD) and mammals (Shannon–Wiener, Funct MPD, Funct FNND, PD, Phylo MPD, Phylo MNTD, and Phylo SES PD), which varied across different seasons. These findings emphasize that plant diversity and seasonal changes are closely related to the diversity and community structure of birds and mammals. They provide theoretical support for the role of habitat vegetation and seasonal dynamics in maintaining the stability and functioning of montane animal ecosystems, offering important insights for addressing habitat fragmentation and species migratory behavior.

## Simple Summary

Elucidating the patterns and processes of montane wildlife diversity is vital for biodiversity conservation, yet in recent decades, widespread plantations of economic forests and tourism development have increasingly threatened their habitats with degradation and destruction. Human activities such as deforestation and illegal hunting further accelerate the loss of wildlife diversity in these regions. Using long-term monitoring data obtained by infrared-triggered cameras, this study examined the relationships between the diversity and community structure of plants, terrestrial birds, and mammals in the subtropical region of eastern China. It also investigated differences in animal diversity and altitudinal migration under different seasonal variations. Our findings showed that habitat plant diversity was correlated with bird and mammal diversity at both taxonomic and phylogenetic levels. Seasonal variation significantly influenced the diversity of birds and mammals across taxonomic, functional, and phylogenetic dimensions. Mammals showed greater sensitivity to plant diversity than birds. These results underscore the importance of adopting multidimensional approaches to understand the complex relationships among habitat vegetation, animal community diversity, and seasonal dynamics, providing new perspectives for future wildlife conservation in the montane region.

## 1. Introduction

Montane regions, which make up about 25% of the Earth’s terrestrial land area (excluding Antarctica), are home to nearly 87% of the world’s species of amphibians, birds, and mammals. These areas are not only critical habitats for a vast proportion of the Earth’s vertebrate species, but they also serve as hotspots for biodiversity, with some endemic species primarily restricted to these montane environments [1]. However, in recent decades, the large-scale cultivation of montane plantation forests and the development of mountain tourism have not only led to a sharp increase in rates of habitat destruction and degradation but also intensified human–wildlife conflicts [2,3]. Particularly notable are activities such as deforestation and illegal hunting, which may influence the functional diversity of animal communities, thereby exacerbating biodiversity loss and impairing the restoration of ecosystem functions [4].

Acting as key components of forest ecosystems, wild animals serve as vital connectors between habitats and ecosystems through three principal roles: resource linkers (facilitating energy flow), genetic linkers (enabling seed dispersal and pollination), and process linkers (via cross-habitat foraging and prey dynamics) [5], thus influencing the functions and structures of ecosystems [6]. For example, long-distance transport by birds, relative to other dispersal methods, also helps break the geographical isolation of different community vegetation [7]. New calculations indicate that 90% of flowering plant species are animal-pollinated [8]. However, driven by human activities, habitat fragmentation and land-use changes may jointly impair both flower-visitation frequency and animal movement capacity [9,10]. The disruption of plant–pollinator interactions, along with the resulting loss of genetic diversity (including heterozygosity and allelic diversity), could be one of the most severe impacts on ecosystem stability [11]. Limited previous research has found that plant richness is associated with bird richness, the phylogenetic structure (Phylo NRI), and functional structure (Funct NRI) [12]. However, there was no significant correlation between the plant Shannon–Wiener (such as trees, shrubs, and herbs) and bird diversity metrics [13]. Overall, future research into the multidimensional relationships between habitat plant and animal communities is crucial for understanding how montane forests maintain ecosystem function and sustainability.

Moreover, animals usually have activity rhythms corresponding to seasonal changes [14]. These variations are reflected in the physiological ecology and migratory behavior traits of animals [15,16]. For example, seasonal migration not only allows animals to acquire new breeding territories to improve breeding success [17] but also helps in avoiding survival pressures during resource-scarce seasons [18]. Therefore, a conspicuous season–diversity relationship is often observed in seasonal environments [14,19]. Previous studies have found significant seasonal variations in the Shannon–Wiener index of urban birds [20]. The species richness of typical montane birds shows a mid-peak pattern across seasons [21]. Although the influence of seasonality on biogeographic patterns of species diversity (such as the Shannon–Wiener index) has been widely acknowledged, our understanding of how seasonality affects other dimensions of biodiversity, such as functional and phylogenetic diversity, remains limited. The limited focus on how species diversity responds to seasonal changes overlooks the critical role of seasonal changes in ecosystem functioning [22]. Sparse studies suggest that seasonal turnover of functional diversity increases with the intensity and predictability of seasonality [23]. Therefore, research on functional and phylogenetic diversity based on species’ physiological morphology and life history characteristics is crucial for understanding how communities withstand long-term environmental changes, maintain ecosystem functions, and evolve, as well as for understanding community evolution and assembly mechanisms [22,24].

The study of montane animal diversity provides an important theoretical foundation for wildlife conservation efforts [21,25,26,27]. However, research on the impact of multidimensional plant diversity levels and seasonal changes on the diversity of birds and mammals is relatively scarce [12,13,23]. The complexity of mountains, arising from immense variation in geology, climate, and species evolution, is strongly associated with high biodiversity. Consequently, mountain formation acts as a critical generator, cradle, and long-term reservoir for species [28]. Therefore, research on the taxonomic, functional, and phylogenetic diversity of wildlife, as well as community structure, is crucial for a better understanding of the life habits of montane animals. In this study, we conducted a four-year dynamic monitoring of ground-dwelling birds and mammals and investigated the vegetation composition of animal habitats in the subtropical region of eastern China. We aimed to explore the following three issues: (1) to clarify the role of phylogenetic relatedness in habitat use, we assessed whether birds and mammals with closer phylogenetic relationships visited the same habitat more frequently; (2) to understand the habitat basis of animal communities, we evaluated the correlation between plant diversity and the corresponding diversity and community structure of birds and mammals; and (3) to reveal the temporal dynamics of montane wildlife, we examined how seasonal changes affected the diversity, community structure, and mean altitudinal migration patterns of birds and mammals.

## 2. Materials and Methods

### 2.1. Study Area

**Figure 1:**
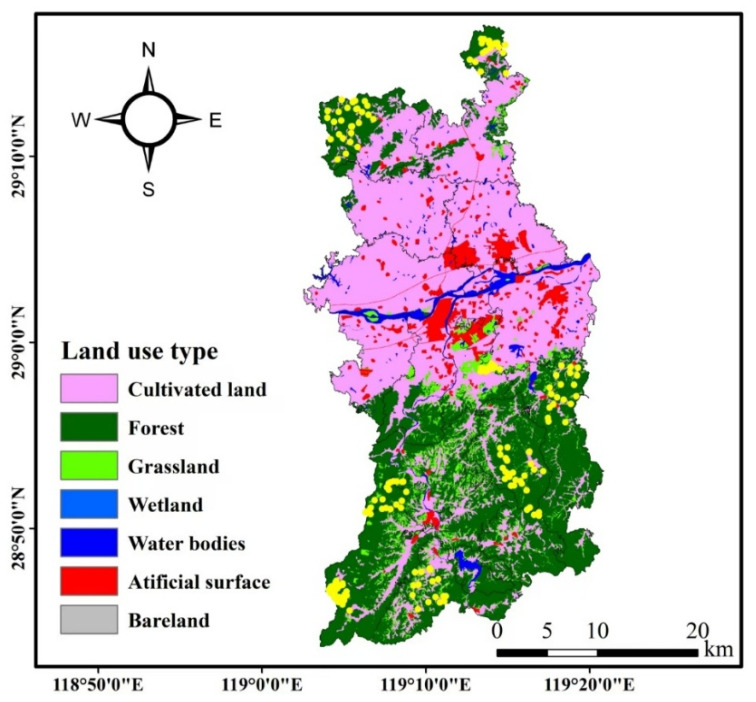
Distribution map of 203 infrared-triggered camera sites in Longyou County, Zhejiang Province, China; The yellow circles represent the camera sites.

**Figure 2:**
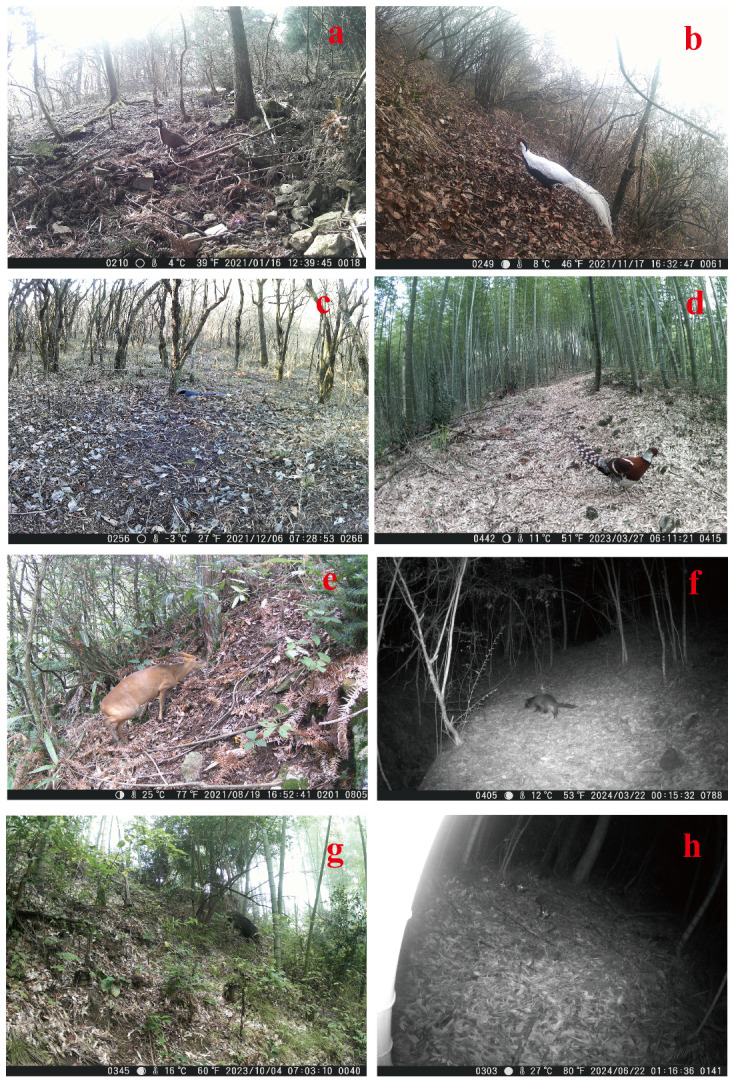
Some images of the landscapes of birds and mammals; (**a**) *Pucrasia macrolopha*; (**b**) *Lophura nycthemera*; (**c**) *Urocissa erythroryncha*; (**d**) *Syrmaticus ellioti*; (**e**) *Muntiacus reevesi*; (**f**) *Paguma larvata*; (**g**) *Sus scrofa*; (**h**) *Arctonyx collaris*.

The study was conducted in Longyou County (28°44′–29°17′ N, 119°02′–119°20′ E), Zhejiang Province, China. The county spans an elevation of 33–1442 m over an area of 1143 km^2^, exhibiting an elongated profile that measures 61.5 km east–west and 29.4 km north–south. The region experiences a subtropical monsoon climate with distinct wet and dry seasons [29], a mean annual temperature of 15.1 °C, and a mean annual precipitation of 1703.9 mm [30]. For the purpose of this study, the months were assigned to four climatic seasons: spring (March–May), summer (June–August), autumn (September–November), and winter (December–February) [31]. We deployed a total of 203 H805 infrared-triggered cameras, manufactured by Hong’e Electronic Technology Co., Ltd. in Kunshan, China. The infrared-triggered camera technology provides more repeatable, comparable, and sustainable data on regional fauna than traditional inventory surveys. It is particularly effective for surveying medium-to-large mammals and diurnal small mammals. For bird surveys, the method is well-suited for ground-dwelling birds, especially pheasants [32]. These cameras were continuously deployed at fixed locations across 29 transects for a four-year period (January 2021–December 2024) to conduct long-term dynamic monitoring of ground-dwelling birds and mammals (Figure 1). For the purpose of this study, the ground-dwelling birds and mammals specifically refer to species monitored by infrared-triggered cameras (Figure 2). We adopted a random deployment strategy for the infrared-triggered cameras, based on the local topography and vegetation types, ensuring a minimum distance of 200 m between any two adjacent cameras [33]. The camera transects were designed to ensure coverage of all major forest types identified within the study area (e.g., broad-leaved, mountain shrub, Moso bamboo, and *Cunninghamia lanceolata* forests), thereby sampling the range of animal habitats present [34]. Camera installations were secured to trees approximately 0.5 m above ground, with the lens oriented away from direct sunlight. The parameter settings were continuous shooting mode (3 photos + 15 s video per trigger), a 1 s interval between triggers, and medium sensitivity [33]. The images of the same animal taken by an infrared-triggered camera within 30 min were recorded as a single independent record (frequency of one visit) [35].

### 2.2. Data Collection

The identification and classification of habitat plants and animals were conducted with reference to A Field Guide to the Birds of China [36], A Guide to the Mammals of China [37], the Species 2000 China Node (http://col.especies.cn/, accessed on 1 October 2025), the Flora of China (https://www.iplant.cn/, accessed on 1 October 2025), and FlowerMate 2.0 [38]. Species lists for birds and mammals were compiled (Tables S1 and S2). Furthermore, vegetation at all 203 camera sites was surveyed using a nested-plot design: a 20 × 20 m plot for trees (DBH: diameter at breast height; DBH > 2 cm), three 5 × 5 m subplots for shrubs, and five 1 × 1 m subplots for herbs. Among the herbaceous plants, we grouped those with woody stems into the shrub category, while those with herbaceous stems remained classified as herbaceous plants.

In addition, we collected some functional traits relevant to mammals and birds from previous research. For mammals, the main traits included body mass, diet (categorized as herbivore, omnivore, or carnivore), and habitat breadth (the number of distinct suitable levels inhabited by species) from a dataset in China [39]. For birds, the specific traits were body mass, habitat preference, and trophic niche from a global dataset [40]. Collectively, these functional traits reflect the morphology, ecology, and adaptive strategies of the animals [41,42,43]. For plants, we applied the plant functional trait (leaf area: SLA; leaf dry matter content: LDMC; plant height; leaf nitrogen content) on the TRY data portal (https://www.try-db.org/, accessed on 1 October 2025). Then, we used the *rtry* package in R to process the original data. The TRY Plant Trait Database is a comprehensive global database of plant functional traits from the TRY initiative. This dataset contains standardized measurements of key plant functional traits across multiple species, genera, and families. This dataset includes key traits related to the leaf economics, plant architecture, reproductive strategy, wood anatomy, and chemical composition [44]. Plant functional traits are morphological, physiological, and phenological characteristics that influence plant fitness and ecosystem functioning. These traits result from evolutionary and community assembly processes that are shaped by both abiotic and biotic environmental constraints [44,45,46]. Functional trait data were available for over 70% of the surveyed plant species from the TRY database. For the remaining species with missing trait data, we applied a phylogenetic imputation approach using the *Rphylopars* package. The imputation was performed under Pagel’s lambda (λ) evolutionary model to account for the phylogenetic signal while incorporating both phylogenetic and phenotypic correlations between species and traits. The uncertainty associated with each imputed value was quantified by calculating its 95% confidence interval based on the posterior variance estimated by the model [47].

### 2.3. Data Analysis

We tested the phylogenetic signal in the species degree (number of animal–habitat interaction networks) of plants and animals for both four-year and seasonal networks. We calculated phylogenetic signal using two different methods (*K* statistic, Pagel’s *λ*) [48,49]. The *K* statistic provides the amount of signal relative to that expected under Brownian motion (random walk) on a given phylogeny [48,50]. A *K* value > 1 generally indicates a phylogenetic signal stronger than expected under a Brownian motion model of evolution for the frequency of habitat visitation among species, whereas a *K* value < 1 suggests a phylogenetic signal weaker than expected under the same model. Furthermore, when λ = 0, it indicates that the trait has evolved independently of phylogeny (no phylogenetic signal). When λ = 1, it indicates that the trait has evolved according to a Brownian motion model along the phylogenetic tree. We calculated the *K* statistic, Pagel’s *λ*, and the *p*-value from a randomization test using the ‘phylosig’ function in the R package *phytools* [50,51].

In terms of taxonomic diversity, we utilized the diversity function from the R package *vegan* to calculate species richness (SR) and the Shannon–Wiener index for both plant and animal communities [52]. The Shannon–Wiener index quantifies community diversity by incorporating both species richness and evenness [53]. The range of the Shannon–Wiener index is ≥0. When the value equals 0, it indicates that there is only one species in the community. The higher index values correspond to greater species richness and a more equitable distribution of individuals among species, thereby reflecting increased community diversity. Additionally, we computed three functional diversity indices using the mFD package: functional dispersion (FDis), mean pairwise functional distance (Funct MPD), and mean nearest functional neighbor distance (Funct FNND), used by the *mFD* package in R [54,55,56]. FD usually refers to FDis, which is the weighted deviation from the center of gravity of species in the assemblage. A higher FD (≥0) value indicates that the functional trait differences among species within the community are greater. The Funct MPD (≥0) is the mean weighted distance between all pairs of species, and a higher Funct MPD value indicates that the community is composed of species with significant functional differences. The Funct FNND (≥0) is the weighted distance to the nearest neighbor within the assemblage; a lower Funct FNND value indicates a greater similarity between the species’ functions and suggests a higher likelihood of intense competition. Regarding phylogenetic diversity, we pruned global phylogenetic trees for birds [57] and mammals [58] to our local species lists using the ‘phytools’ package in R [59]. Correspondingly, to assess diversity from a phylogenetic perspective, the phylogenetic diversity (PD), mean pairwise phylogenetic distance (Phylo MPD), and mean nearest phylogenetic taxon distance (Phylo MNTD) were computed using the ‘pd’, ‘mpd’, and ‘mntd’ functions, respectively, from the R package *picante*. The PD (≥0) quantifies the total evolutionary history embodied in an assemblage by summing the branch lengths connecting all species on a phylogenetic tree. The Phylo MPD (≥0) measures the overall phylogenetic relatedness among species as the average phylogenetic distance between all possible species pairs. The Phylo MNTD reflects the degree of local phylogenetic clustering by calculating the average distance from each species to its nearest relative within the assemblage [60]. We also calculated the standardized effect size of PD (Phylo SES PD) using the ‘ses.pd’ function in the package *picante* [60]. The Phylo SES PD reflects whether the observed phylogenetic diversity is higher (>0) or lower (<0) than the random expectation [61].

In the functional and phylogenetic structure, we calculated the nearest taxon index (NTI) and net relatedness index (NRI) based on the mean pairwise phylogenetic and functional taxon distances and mean nearest phylogenetic and functional taxon distances [62]. Notably, to ensure more accurate community structure analyses based on valid animal records captured by infrared-triggered cameras, functional and phylogenetic community structures were implemented in the R package *picante* using the ‘frequency’ null model [60]. Values of NRI and NTI greater than 1.96 indicate significant clustering, whereas values less than −1.96 indicate significant overdispersion [61]. The NRI and NTI were computed using the ‘sesmpd’ and ‘sesmntd’ functions, respectively, from the R package *picante*. The NRI tends to reflect the overall phylogenetic relatedness composition of species within a community, whereas the NTI focuses on local phylogenetic relatedness composition [63]. Values of NRI or NTI > 0 indicate phylogenetic clustering (i.e., species are more closely related than expected by chance), whereas values of NRI or NTI < 0 suggest phylogenetic overdispersion. When values of NRI or NTI = 0, the phylogenetic structure of the community composition resembles that of a randomly assembled community, implying that both environmental and biotic factors collectively maintain the diversity of species structure [64,65].

Finally, the ordinary least squares (OLS) regression is the most direct and classic method for achieving the goal of describing the linear relationship between two continuous variables [66]. We conducted relevant hypothesis tests on the data, followed by OLS analysis to examine the relationship between the plant and animal diversity value in different communities. For birds, the group samples of different seasons under the same metric were from 27 to 124, with an average group sample size of 62, while the group samples under different seasons were from 34 to 150, with an average of 83 group sample sizes for mammals. In order to understand the significant impact of seasonal changes on the level of animal diversity, we first conducted an overall significance test using Kruskal–Wallis [67], followed by Dunn’s test with Bonferroni correction for multiple comparisons to acquire pairwise seasonal differences in diversity parameters [68]. All statistical analyses were performed in R software (version 4.4.0) [69].

## 3. Results

### 3.1. Phylogenetic Signals

**Figure 3:**
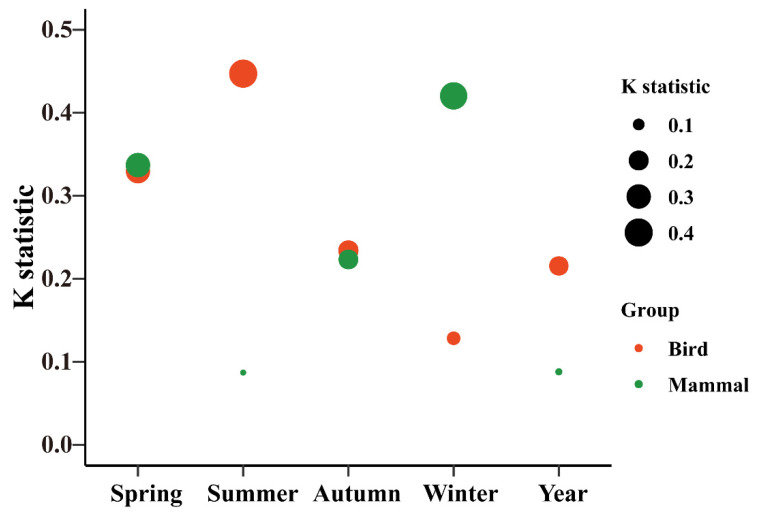
The phylogenetic signal (*K* statistic) in the frequency degree of cumulative animal–habitat interaction networks for four years; The size of circles corresponds to the frequency of animal–habitat interaction; The x-axis represents the total frequency of animals visiting their habitats for four years.

We recorded 72 bird species across 8 orders and 25 families, with a total of 17,088 independent valid photographs (Table S1). The number of independent photographs of birds varied across seasons (spring: 4649; summer: 4579; autumn: 5327; winter: 2533). For mammals, 19 species from 7 orders and 12 families were documented, totaling 27,499 independent valid records (Table S2). Seasonal variation was also observed in the number of independent photographs of mammals (spring: 6466; summer: 7667; autumn: 8144; winter: 5222). In the animal–habitat interaction network, no significant phylogenetic signal was detected by the *K* statistic in the frequency of different birds visiting their habitats for all birds–habitat (*K* = 0.216, *p* > 0.05). However, a significant signal was observed using Pagel’s *λ* (*λ* = 0.528, *p* < 0.05) (Figure 3, Table S3). For all mammals, neither the *K* statistic nor Pagel’s *λ* revealed a significant phylogenetic signal in the frequency of different mammals visiting their habitats. In terms of seasonal variation, significant phylogenetic signals in birds were only found in spring and summer using the *K* statistic, whereas Pagel’s *λ* indicated significant signals in spring, summer, and autumn. For mammals, neither the *K* statistic nor Pagel’s *λ* revealed consistent phylogenetic signals across seasons (Table S3).

### 3.2. Diversity and Community Structure

In the taxonomic diversity (Figure S1), we found that plant SR showed a significant positive correlation with both bird and mammal SR within shared habitats. Regarding functional diversity, no significant correlations were detected between habitat plant and the corresponding functional diversity indices (FD, Funct MPD, and Funct FNND) in the birds or mammals. In the phylogenetic diversity, there was a significant positive correlation between plants and birds as well as mammals (plant PD vs. bird PD, plant Phylo SES PD vs. bird Phylo SES PD, plant MPD vs. mammal MPD, and plant Phylo MNTD vs. mammal Phylo MNTD). In the functional and phylogenetic structure, the plant Funct NRI showed significant positive correlations with the bird Funct NRI. In contrast, the plant Phylo NRI and Phylo NTI demonstrated significant positive correlations with corresponding mammal diversity indices.

**Figure 4:**
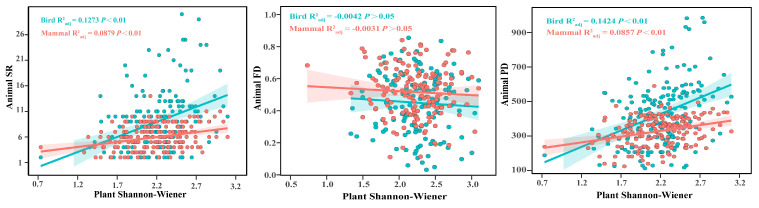
The correlation between habitat plant Shannon–Wiener and animal species richness (SR), functional diversity (FD), and phylogenetic diversity (PD) by an ordinary least squares regression analysis; The shaded areas represent the 95% credible intervals; The dots represent the values of diversity metrics for the same habitat.

In the longitudinal analysis, we examined the associations of plant Shannon–Wiener with bird and mammal diversity metrics within shared habitats (Figure 4 and Figure S2). The following relationships emerged: For species diversity, plant Shannon–Wiener exhibited significant positive correlations with both bird and mammal SR. However, no significant correlations were found between plant Shannon–Wiener and functional diversity indices (FD, Funct MPD, and Funct FNND). Regarding phylogenetic diversity, plant Shannon–Wiener showed a significant positive relationship with the PD and Phylo SES PD of both birds and mammals while showing a significant negative relationship with the Phylo MNTD. In terms of community structure, the significant positive correlations were specifically observed between plant Shannon–Wiener and the mammal Funct NTI and Phylo NTI.

### 3.3. Seasonal Change in Birds and Mammals

**Figure 5:**
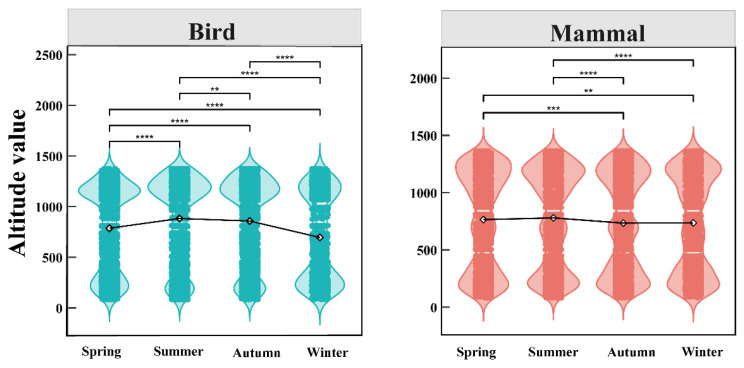
Differences in bird and mammal altitudinal migration across different seasons based on the Kruskal–Wallis test; The diamond symbols represent the average altitude of mammals and birds in different seasons; ** *p* < 0.01, *** *p* < 0.001, **** *p* < 0.0001.

Based on four years of long-term seasonal monitoring data, the analyses of altitudinal migration in birds and mammals were as follows (Figure 5). The altitude for a bird or mammal species was defined as the elevation of the camera-trap location where the species was photographed. For birds, the 17,088 independent valid bird photographs revealed a trend of upward shift in mean altitude from spring to summer, followed by a descending trendline through autumn and winter. Notably, the average altitudes of birds and mammals varied significantly in different seasons (spring: 781 m, summer: 877 m, autumn: 852 m, winter: 691 m). For mammals, the analysis of 27,499 independent valid records showed a more gradual pattern of initial increase followed by decrease from spring to winter; significant seasonal differences in their mean altitudinal migration were also detected (spring vs. autumn/winter; summer vs. autumn/winter) (spring: 764 m, summer: 779 m, autumn: 734 m, winter: 691 m).

**Figure 6:**
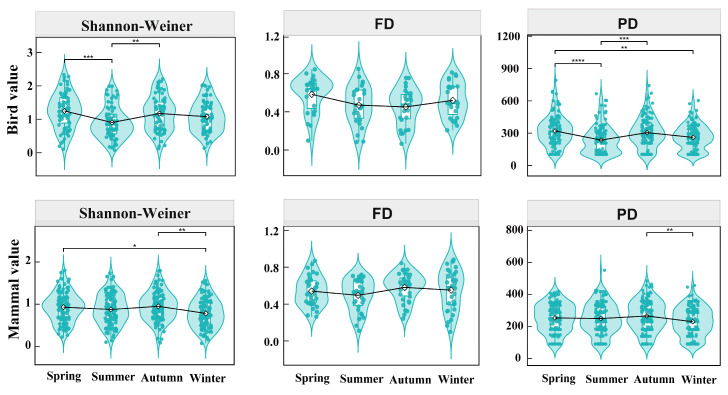
Differences in bird and mammal Shannon–Wiener, functional diversity (FD), and phylogenetic diversity (PD) across different seasons based on the Kruskal–Wallis test; * *p* < 0.05, ** *p* < 0.01, *** *p* < 0.001, **** *p* < 0.0001.

Further analysis demonstrated that both bird and mammal diversity changed seasonally (Figure 6 and Figure S3). Specifically, in terms of species diversity, the significant seasonal differences based on multiple comparisons were found in bird Shannon–Wiener (summer vs. spring/autumn) and mammals (winter vs. spring/autumn). Regarding functional diversity, the significant differences in different seasons were detected in bird Funct FNND (autumn vs. spring/winter), mammal Funct MPD (spring vs. summer/winter; summer vs. autumn/winter), and mammal Funct FNND (spring vs. autumn/winter; summer vs. autumn/winter). In phylogenetic diversity, significant seasonal variations occurred in bird PD (spring vs. summer/winter; summer vs. autumn) and mammal PD (autumn vs. winter), while only mammals showed significant seasonal differences in Phylo MPD (summer vs. winter) and Phylo MNTD (winter vs. spring/summer/autumn). Significant differences in different seasons were found in mammal Phylo SES PD (spring vs. summer/autumn/winter). No significant seasonal differences were found in the functional or phylogenetic structure metrics (Funct NRI, Funct NTI, Phylo NRI, and Phylo NTI) of birds and mammals.

## 4. Discussion

### 4.1. Phylogenetic Signal in Species Frequency Degree

The mutually beneficial relationships between plants and animals are a common and ecologically significant phenomenon in terrestrial ecosystems. For instance, they acquire food resources and disperse seeds, forming intricate networks of interdependence [70]. Previous studies have demonstrated that the phylogenetic relationships of community species were associated with the number of interactions in over one-third of the networks and the specific interacting species in approximately half of them [71]. A strong phylogenetic signal has been observed in species degree and interaction strength for ectomycorrhizal fungi but not for plants [72]. Similarly, weak and non-significant phylogenetic signals were detected in the species degree of plant and bird phylogenies assessed by the *K* statistic and Pagel’s *λ* for both year-round and monthly networks of seed dispersal and pollination interactions [50]. In contrast, our results demonstrate the significant phylogenetic signals (Pagel’s *λ*) in birds across different seasons, except for winter. Additionally, the significant phylogenetic signals (*K* statistic) were observed specifically during the birds’ spring and summer (Table S3). This methodological discrepancy may be due to stronger selective pressures during spring and autumn for birds, which reflects phylogenetic niche conservatism [50,73,74]. However, no significant signal (Pagel’s *λ*; *K* statistic) was detected in mammals. The potential explanation for this finding is that mammals exhibit lower mobility across regional montane forests compared to birds, resulting in less distinct variation in species composition.

### 4.2. The Interaction Relationship Between Plants and Animals

In the present study, comparing plant and animal diversity across shared habitats (Figure S1), the correlation analyses revealed that plant SR was significantly positively correlated with both bird and mammal SR. This finding aligns with several previous studies [12,26,75]. The habitat heterogeneity hypothesis posits that areas with greater habitat diversity tend to support higher species diversity by providing a wider variety of ecological niches, more opportunities for geographic isolation that promote speciation, and additional refuges for species, thereby facilitating species coexistence [76,77,78,79]. Notably, our results showed significant positive correlations between plant PD and bird PD, as well as between plant Phylo SES PD and bird Phylo SES PD. However, plant Phylo MPD, Phylo MNTD, Phylo NRI, and Phylo NTI were significantly positively correlated only with corresponding mammal diversity metrics. This indicates that changes in plant diversity have a greater impact on mammal diversity than on bird diversity. This pattern suggests that mammals have reduced mobility and smaller home ranges than birds at a regional scale. Consequently, the mammalian diversity within a given habitat is more strongly dependent on the diversity of local plant resources.

Furthermore, our results demonstrated that the plant Shannon–Wiener index was significantly positively correlated with both bird and mammal SR (Figure 4). A plausible explanation is that a higher plant Shannon–Wiener index, through increased vegetation structural complexity, provides greater food resources for herbivores, thereby enhancing bird and mammal richness [80]. Moreover, as species diversity increases, communities become more vertically stratified [81], which enhances structural complexity (such as forest canopy), which further promotes species diversity in birds and mammals [82,83]. Our study further revealed that the plant Shannon–Wiener index had significant positive correlations with both bird and mammal PD and Phylo SES PD while exhibiting significant negative correlations with Phylo MNTD (Figure S2 and Figure 4). Additionally, the plant Shannon–Wiener index showed no significant correlation with phylogenetic and functional NRI of birds and mammals (Figure S2), consistent with previous findings [13]. This may be attributed to the close phylogenetic relationships among species at the regional scale, resulting in weak or non-significant effects of community assembly processes [84]. Previous research also reported no significant association between plant Shannon–Wiener and avian functional diversity metric (FD) in southwestern China [13]. However, we found that the plant Shannon–Wiener index was significantly positively correlated with both phylogenetic and functional NTI of mammals (Figure S2). This suggests that in plant–animal interaction networks, it is crucial to consider not only plant richness but also abundance in providing sufficient food resources for preferred animal species. In summary, our findings underscore the importance of a multidimensional approach to analyzing biodiversity and caution against relying solely on any single diversity metric [62].

### 4.3. Seasonal Variations in Animal Biodiversity and Migration

The food-limitation hypothesis posits that seasonal variations are closely linked to the abundance of forest food resources, as animals can track changes in resource availability within their habitats [85]. Consequently, fluctuations in food resource abundance influence the dynamics of bird communities, including species richness and abundance [50,86]. Furthermore, seasonal variations in food resources are considered a major driver of altitudinal migration in ungulates [85]. For instance, studies in temperate regions have shown that abundant food resources and predation risk during spring and summer often drive ungulates and birds to higher elevations [87,88,89], while winter precipitation, snow cover, and food scarcity lead to downward shifts to lower altitudes [90,91]. Although the study sites had a relatively low overall altitude, our four-year extensive monitoring data clearly demonstrated that both birds and mammals exhibited significant seasonal differences in their mean altitudinal migration (Figure 5). These seasonal altitudinal migration characteristics provide a valuable context for studying variations in animal diversity across communities [85]. Previous research has shown that the diversity of bird species in urban gardens, as indicated by the Shannon–Wiener index, varies seasonally, with higher values during spring and autumn due to the presence of migrating species [20]. Our findings also demonstrate that seasonal changes significantly affected only bird Shannon–Wiener, Funct FNND, and PD, whereas mammals exhibited more extensive significant responses across taxonomic (Shannon–Wiener), functional (Funct MPD and Funct FNND), and phylogenetic diversity (Phylo MPD, Phylo MNTD, and Phylo SES PD) metrics (Figure 6 and Figure S3). This may be attributed to birds’ broader resource selectivity and higher mobility [50,71], as well as the fact that larger-bodied mammals require more substantial food resources, leading to more frequent mutualistic interactions between mammals and their habitats at the regional scale.

### 4.4. Conservation Implications

Historically, forest managers have primarily focused on the richness and abundance of rare animal species, often overlooking the critical role of wildlife phylogenetic and functional diversity in maintaining ecosystem stability [62]. Our study reveals that closely related bird species exhibit higher visitation frequencies to the same habitats and that habitat vegetation complexity significantly correlates with animal diversity. Furthermore, seasonal variations amplify these impacts on the community diversity of birds and mammals. Therefore, we emphasize that greater attention to the floristic composition of animal habitats will promote the diversity of forest animal communities. Notably, an increase in insectivorous birds can, through negative feedback mechanisms, contribute to the control of forest pests and diseases. Despite the non-significant phylogenetic signal in the habitat visit frequency of closely related mammals, an increase in mammal richness was associated with enhanced bird diversity within the community. For instance, the research suggested that the wild boar might have consistent positive impacts on habitat utilization by other sympatric species in the Liupanshan Mountains, highlighting their role in maintaining species assemblages in well-protected nature reserves [92]. This underscores the importance of dedicating greater attention to multidimensional diversity dynamics in forest bird and mammal communities.

Moreover, our findings indicate that the phylogenetic background of plant communities is either positively or neutrally associated with bird and mammal diversity in shared habitats. Under previous national forestry policies, montane regions in eastern China often prioritized planting tree species valued for ornamental appeal or high survival rates (such as *Phoebe sheareri*) to obtain policy-based subsidies. Consequently, future implementation of policies promoting scientifically informed tree species mixtures in montane regions will enhance bird and mammal diversity and community structure. Our study also demonstrates that the plant Shannon–Wiener index (incorporating richness and abundance) is equally crucial for supporting the dietary preferences and ensuring adequate food resources for birds and mammals. Long-term monitoring data from this study confirm significant seasonal differences in the altitudinal migration patterns of birds and mammals in montane forests, which further accentuate seasonal fluctuations in community animal diversity. As our study area spans multiple montane regions, these results suggest that conserving adequate, large, and interconnected habitat patches is essential for protecting biodiversity and maintaining overall biological integrity [86]. In this study, we used infrared-triggered camera monitoring to investigate the effects of habitat plant diversity and seasonality on wildlife. However, our analysis incorporated a limited number of predictors, resulting in a model of relatively low complexity. In future research, we plan to include additional variables, such as forest type, altitudinal gradient, and human disturbance, into the model. This will allow us to assess their correlation with wildlife activity and analyze the relative importance of each factor. Identifying these key drivers is essential for developing effective strategies to enhance wildlife diversity in China’s subtropical montane forests.

## 5. Conclusions

Our research examined how habitat plants and seasonal variations shaped animal diversity in Longyou County, eastern China. We detected significant phylogenetic signals in the frequency degree of bird–habitat across certain seasons, as measured by both Pagel’s *λ* and the *K* statistic, whereas no such signal was detected in mammals across different seasons. We also found that the results of plant and animal diversity showed differential associations for birds and mammals, rather than uniform patterns. Furthermore, we found that the seasonal changes influenced the mean altitudinal migration and diversity of birds and mammals. However, no significant differences were observed in the functional structures (Funct NRI and Funct NTI) and phylogenetic structures (Phylo NRI and Phylo NTI) of birds and mammals. These findings show that adopting a multidimensional diversity approach is helpful to understand the impact of habitat vegetation diversity and seasonal variations on wildlife. Moreover, given that climate change is prolonging seasons and amplifying extreme weather, the ecological dynamics of animal diversity levels may become more pronounced. Therefore, this approach becomes important for predicting and mitigating long-term impacts on montane biodiversity.

## Data Availability

The datasets analyzed during the current study are available from the corresponding author upon reasonable request.

## Supplementary Materials

The following supporting information can be downloaded at https://www.mdpi.com/article/10.3390/ani16020215/s1, Figure S1: Bird species list recorded by infrared-triggered cameras across sampling sites; Figure S2: Mammal species list recorded by infrared-triggered cameras across sampling sites; Figure S3: Amount and significance of phylogenetic signal of frequency degree of animal-habitat interaction networks for both different seasons and four years used *K* statistic and Pagel’s *λ*; Table S1: The correlation between plant diversity and its corresponding animal diversity and community structure; Table S2: The correlation between plant Shannon-Wiener diversity and animal diversity and community structure; Table S3: The differences in bird and mammal diversity and community structure across different seasons based on Kruskal-Wallis test.
