# Supplementary material for: Plant Diversity and Seasonal Variation Drive Animal Diversity and Community Structure in Eastern China
Source: Animals (Basel). 2026 Jan 11;16(2):215. doi: 10.3390/ani16020215 (PMC12837589; doi:10.3390/ani16020215)
Supplement: Supplementary file 1 [file animals-16-00215-s001.zip › animals-4044559-supplementary.pdf]

Plant Diversity and Seasonal Variation Drive Animal Diversity and Community Structure in Eastern China

Supplementary materials

Table S1. Bird species list recorded by infrared-triggered cameras across sampling sites

Order	Family	Genus	Species
Accipitriformes	Accipitridae	*Accipiter*	*Accipiter trivirgatus*
Accipitriformes	Accipitridae	*Accipiter*	*Accipiter virgatus*
Accipitriformes	Accipitridae	*Nisaetus*	*Nisaetus nipalensis*
Accipitriformes	Accipitridae	*pilornis*	*Spilornis cheela*
Charadriiformes	Scolopacidae	*Scolopax*	*Scolopax rusticola*
Columbiformes	Columbidae	*Chalcophaps*	*Chalcophaps indica*
Columbiformes	Columbidae	*Spilopelia*	*Spilopelia chinensis*
Columbiformes	Columbidae	*Streptopelia*	*Streptopelia orientalis*
Cuculiformes	Cuculidae	*Clamator*	*Clamator coromandus*
Galliformes	Phasianidae	*Arborophila*	*Arborophila gingica*
Galliformes	Phasianidae	*Bambusicola*	*Bambusicola thoracicus*
Galliformes	Phasianidae	*Lophura*	*Lophura nycthemera*
Galliformes	Phasianidae	*Pucrasia*	*Pucrasia macrolopha*
Galliformes	Phasianidae	*Syrmaticus*	*Syrmaticus ellioti*
Galliformes	Phasianidae	*Tragopan*	*Tragopan caboti*
Passeriformes	Aegithalidae	*Aegithalos*	*Aegithalos concinnus*
Passeriformes	Alcippeidae	*Alcippe*	*Alcippe davidi*
Passeriformes	Alcippeidae	*Alcippe*	*Alcippe hueti*
Passeriformes	Corvidae	*Corvus*	*Corvus frugilegus*
Passeriformes	Corvidae	*Dendrocitta*	*Dendrocitta formosae*
Passeriformes	Corvidae	*Garrulus*	*Garrulus glandarius*
Passeriformes	Corvidae	*Urocissa*	*Urocissa erythroryncha*
Passeriformes	Emberizidae	*Emberiza*	*Emberiza elegans*
Passeriformes	Emberizidae	*Emberiza*	*Emberiza spodocephala*
Passeriformes	Emberizidae	*Emberiza*	*Emberiza tristrami*
Passeriformes	Fringillidae	*Fringilla*	*Fringilla montifringilla*
Passeriformes	Laniidae	*Lanius*	*Lanius cristatus*
Passeriformes	Leiothrichidae	*Garrulax*	*Garrulax canorus*
Passeriformes	Leiothrichidae	*Garrulax*	*Garrulax monileger*

Order	Family	Genus	Species
Passeriformes	Leiothrichidae	*Ianthocincla*	*Ianthocincla cineracea*
Passeriformes	Leiothrichidae	*Leiothrix*	*Leiothrix lutea*
Passeriformes	Leiothrichidae	*Pterorhinus*	*Pterorhinus pectoralis*
Passeriformes	Leiothrichidae	*Pterorhinus*	*Pterorhinus perspicillatus*
Passeriformes	Motacillidae	*Anthus*	*Anthus hodgsoni*
Passeriformes	Muscicapidae	*Calliope*	*Calliope calliope*
Passeriformes	Muscicapidae	*Enicurus*	*Enicurus leschenaulti*
Passeriformes	Muscicapidae	*Larvivora*	*Larvivora cyane*
Passeriformes	Muscicapidae	*Larvivora*	*Larvivora sibilans*
Passeriformes	Muscicapidae	*Monticola*	*Monticola gularis*
Passeriformes	Muscicapidae	*Myophonus*	*Myophonus caeruleus*
Passeriformes	Muscicapidae	*Phoenicurus*	*Phoenicurus auroreus*
Passeriformes	Muscicapidae	*Tarsiger*	*Tarsiger cyanurus*
Passeriformes	Paradoxornithidae	*Neosuthora*	*Neosuthora davidiana*
Passeriformes	Paradoxornithidae	*Psittiparus*	*Psittiparus gularis*
Passeriformes	Paradoxornithidae	*Sinosuthora*	*Sinosuthora webbiana*
Passeriformes	Paridae	*Machlolophus*	*Machlolophus spilonotus*
Passeriformes	Paridae	*Parus*	*Parus minor*
Passeriformes	Pittidae	*Pitta*	*Pitta nympha*
Passeriformes	Pycnonotidae	*Hemixos*	*Hemixos castanonotus*
Passeriformes	Pycnonotidae	*Hypsipetes*	*Hypsipetes leucocephalus*
Passeriformes	Pycnonotidae	*Ixos*	*Ixos mcclellandii*
Passeriformes	Pycnonotidae	*Pycnonotus*	*Pycnonotus sinensis*
Passeriformes	Pycnonotidae	*Spizixos*	*Spizixos semitorques*
Passeriformes	Scotocercidae	*Abroscopus*	*Abroscopus albogularis*
Passeriformes	Sturnidae	*Spodiopsar*	*Spodiopsar sericeus*
Passeriformes	Timaliidae	*Cyanoderma*	*Cyanoderma ruficeps*
Passeriformes	Timaliidae	*Erythrogenys*	*Erythrogenys swinhoei*
Passeriformes	Timaliidae	*Pomatorhinus*	*Pomatorhinus ruficollis*
Passeriformes	Turdidae	*Geokichla*	*Geokichla citrina*
Passeriformes	Turdidae	*Geokichla*	*Geokichla sibirica*
Passeriformes	Turdidae	*Turdus*	*Turdus cardis*
Passeriformes	Turdidae	*Turdus*	*Turdus eunomus*
Passeriformes	Turdidae	*Turdus*	*Turdus hortulorum*
Passeriformes	Turdidae	*Turdus*	*Turdus mandarinus*
Passeriformes	Turdidae	*Turdus*	*Turdus obscurus*
Passeriformes	Turdidae	*Turdus*	*Turdus pallidus*
Passeriformes	Turdidae	*Zoothera*	*Zoothera aurea*
Passeriformes	Zosteropidae	*Staphida*	*Staphida torqueola*
Passeriformes	Zosteropidae	*Zosterops*	*Zosterops simplex*
Piciformes	Picidae	*Blythipicus*	*Blythipicus pyrrhotis*
Piciformes	Picidae	*Picus*	*Picus canus*
Strigiformes	Strigidae	*Otus*	*Otus lettia*

Table S2. Mammal species list recorded by infrared-triggered cameras across sampling sites

Order	Family	Genus	Species
Cetartiodactyla	Bovidae	*Capricornis*	*Capricornis milneedwardsii*
Pholidota	Manidae	*Manis*	*Manis pentadactyla*
Cetartiodactyla	Cervidae	*Elaphodus*	*Elaphodus cephalophus*
Cetartiodactyla	Cervidae	*Muntiacus*	*Muntiacus crinifrons*
Eulipotyphla	Erinaceidae	*Erinaceus*	*Erinaceus amurensis*
Primates	Cercopithecidae	*Macaca*	*Macaca mulatta*
Carnivora	Mustelidae	*Mustela*	*Mustela sibirica*
Rodentia	Sciuridae	*Callosciurus*	*Callosciurus erythraeus*
Rodentia	Hystricidae	*Hystrix*	*Hystrix brachyura*
Rodentia	Sciuridae	*Tamiops*	*Tamiops swinhoei*
Carnivora	Felidae	*Prionailurus*	*Prionailurus bengalensis*
Carnivora	Mustelidae	*Mustela*	*Mustela kathiah*
Lagomorpha	Leporidae	*Lepus*	*Lepus sinensis*
Carnivora	Mustelidae	*Melogale*	*Melogale moschata*
Carnivora	Mustelidae	*Arctonyx*	*Arctonyx collaris*
Carnivora	Prionodontidae	*Paguma*	*Paguma larvata*
Cetartiodactyla	Suidae	*Sus*	*Sus scrofa*
Rodentia	Sciuridae	*Dremomys*	*Dremomys pernyi*
Cetartiodactyla	Cervidae	*Muntiacus*	*Muntiacus reevesi*

Table S3. Amount and significance of phylogenetic signal of frequency degree of animal-habitat interaction networks for both different seasons and four years used *K* statistic and Pagel's λ. Group: The types of animal-habitat interaction networks. Total: the total frequency degree of the animal-habitat interaction networks. LR: Likelihood Ratio.

Group	Total	*K* statistic	P	Pagel's λ	LR	*P*
Bird spring-habitat	4643	0.330	0.045	0.440	6.194	0.013
Bird summer habitat	4570	0.447	0.02	0.551	10.739	0.001
Bird autumn- habitat	7319	0.234	0.29	0.347	5.090	0.024
Bird winter - habitat	2527	0.128	0.909	0.242	1.601	0.206
All birds - habitat	17084	0.216	0.195	0.528	13.746	0.000
Mammal spring - habitat	6466	0.337	0.24	0.000	-0.000	1
Mammal summer- habitat	7666	0.087	0.986	0.000	-0.001	1
Mammal autumn - habitat	8143	0.223	0.716	0.000	-0.001	1
Mammal winter - habitat	5221	0.420	0.204	0.000	-0.000	1
All mammals - habitat	27498	0.088	0.985	0.000	-0.001	1

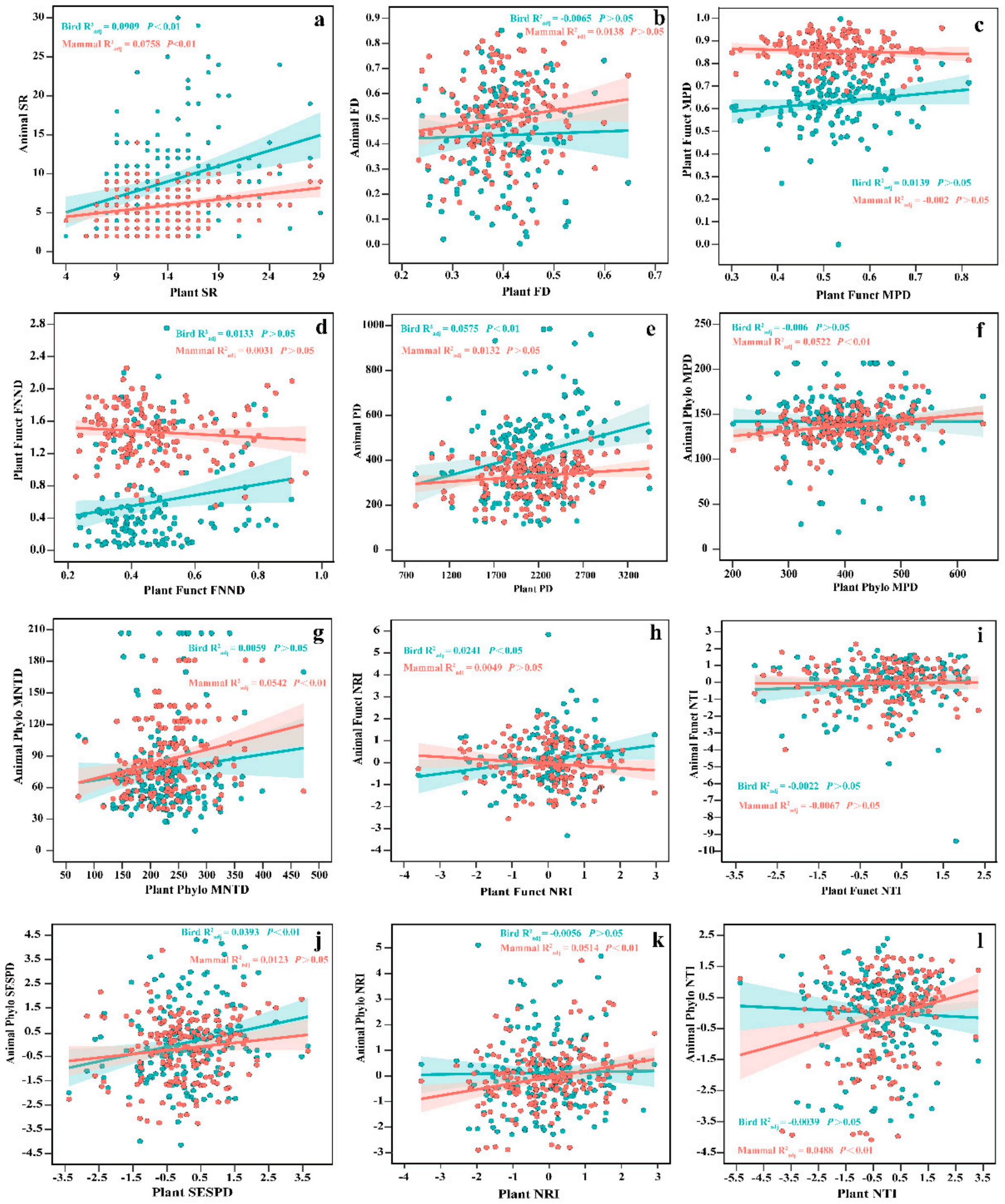

Figure S1. The correlation between plant diversity and its corresponding animal diversity and community structure; The shaded areas represent the 95% credible intervals from an ordinary least squares regression analysis. The dots represent the values of diversity metrics for the same habitat.

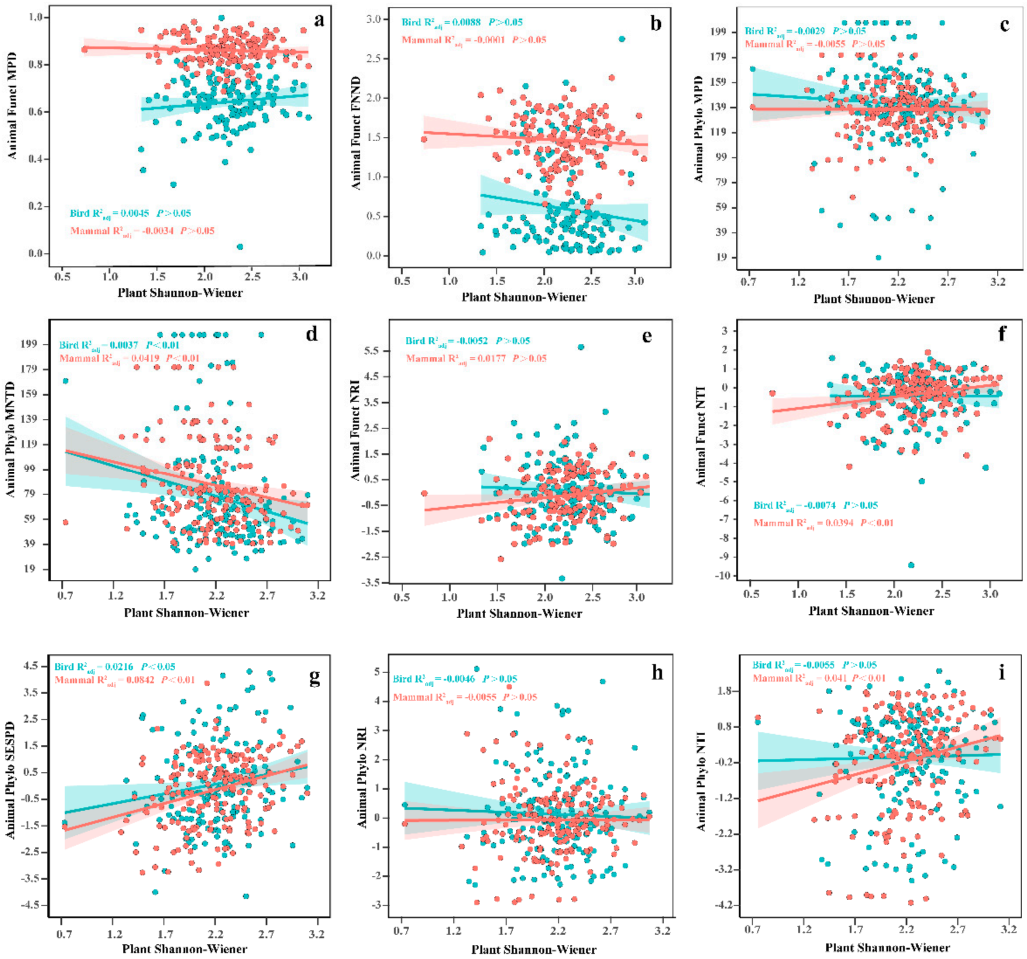

Figure S2. The correlation between plant Shannon-Wiener diversity and animal diversity and community structure; The shaded areas represent the 95% credible intervals from anthe shaded areas represent the 95% credible intervals; The dots represent the values of diversity metrics for the same habitat.

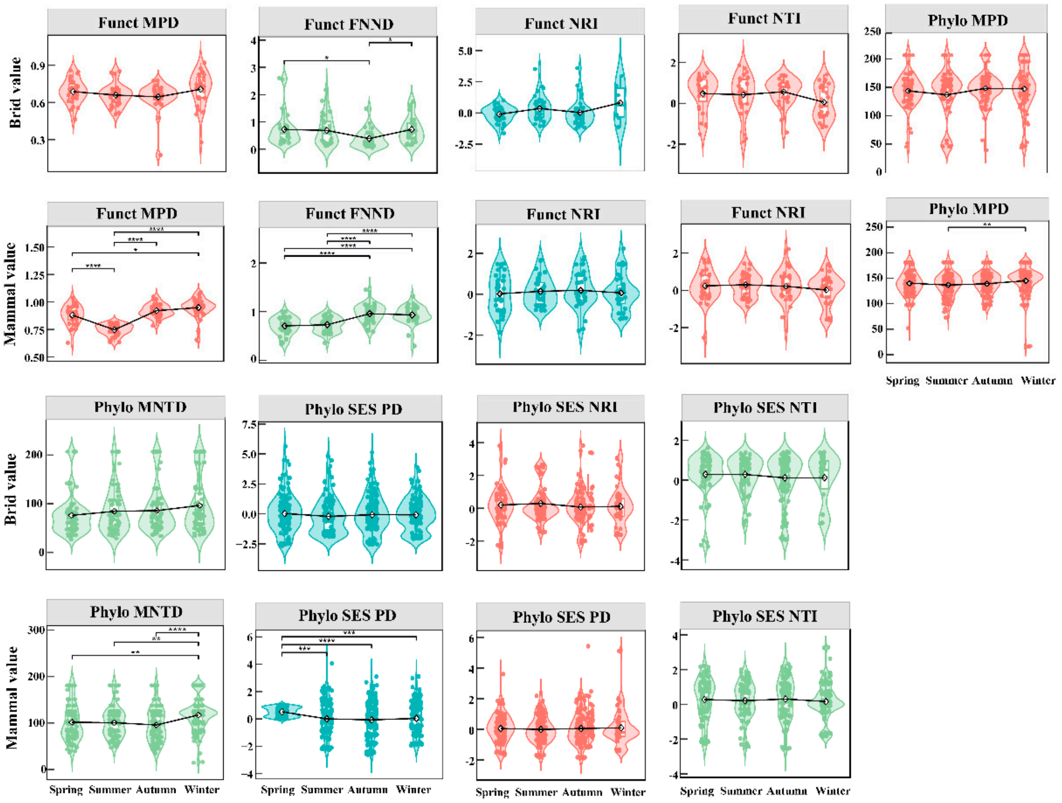

Figure S3. The differences in bird and mammal diversity and community structure across different seasons based on Kruskal-Wallis test; *$p < 0.05$, **$p < 0.01$, *** $p < 0.001$, **** $p < 0.0001$.
